# Quality assurance of functional food products using physical field-assisted freezing

**DOI:** 10.3389/fnut.2026.1762441

**Published:** 2026-04-09

**Authors:** Nikita Tyutkov, Elena Lemeshonok, Angelina Sorokina, Irina Aleksandrova, Victoria Ilina, Artem Lepeshkin, Denis Baranenko

**Affiliations:** 1 International Research Centre “Biotechnologies of the Third Millennium” (Bio3M), ITMO University, Saint-Petersburg, Russia; 2 Zhengzhou Research Institute, Harbin Institute of Technology, Henan, Zhengzhou, China

**Keywords:** electromagnetic radiation, freezing rate, functional food freezing, ice crystal size, microwave assisted freezing, nucleation, ultrasound-assisted freezing

## Abstract

The quality of functional food products depends on the content of biologically active compounds throughout their shelf life. Freezing provides a significant increase in the shelf life of food products. At the same time, conventional food preservation by freezing appears to diminish the content of functional ingredients due to food structure damage and oxidation. This leads to significant losses of biologically active substances and a reduction in the product’s biological activity. Depending on the type of biologically active substance these losses can reach 80%. Enhancing functional ingredients stability is suggested to be achieved using field-assisted freezing techniques. Electromagnetic field-assisted freezing improves retention of L-ascorbic acid in comparison with conventional methods, while ultrasound-assisted freezing improves total phenol retention by 15%, minimizes drip loss, and reduces lipid oxidation. Reported effects vary substantially with processing settings, and evidence for functional food products is still sparse, calling for ingredient- and matrix-specific studies.

## 1 Introduction

Low storage temperatures preserve sensory and nutritional properties of foods. Freezing is widely used as it slows chemical reactions and inhibits microbial growth (1, 2). Freeze damage in cellular matrices remains a key issue. Common mitigation approaches include advanced freezing methods (high freezing rate, ultrasonic or electromagnetic treatment) and pre-freezing infusion of cryoprotectants such as CaCl_2_, sucrose, pectin and minerals. Fast freezing generates many fine intracellular and extracellular ice crystals, reducing water migration and membrane damage (3). Fast freezing methods, including cryogenic techniques, typically produce smaller crystals (4) but are energy-intensive (5, 6). To improve quality while reducing energy use, alternative technologies have been developed such as high-pressure freezing, electrically or magnetically assisted freezing, microwave-assisted freezing, ultrasound-assisted freezing, and CO_2_ infusion immersion freezing. These aim to create small, uniformly distributed crystals, and field-assisted freezing enhances nucleation and crystallization (7).

Functional foods are foods or dietary components that may provide a health benefit beyond basic nutrition. Such products contain ingredients that provide functional effects (polyunsaturated fatty acids, antioxidants, vitamins, minerals, probiotics and prebiotics), which may degrade during freezing. The impact of field-assisted freezing on food quality has been widely studied, including reviews on ultrasound and electromagnetic methods and their effects on freezing efficiency and general product quality (7, 8). However, a focused analysis specifically addressing field-assisted freezing from the perspective of functional foods, where the primary objective is the preservation and stability of functional ingredients rather than only physical quality attributes, is still lacking. The present review aims to bridge this gap by systematically examining how the current field-assisted freezing technologies may influence functional foods and provide preservation of functional components (namely, polyunsaturated fatty acids, antioxidants, and vitamins). Their eventual advancement and adjustment for freezing in the food industry has also been examined.

## 2 Ice nucleation and ice size: the first step towards freezing and its challenges

In the food-freezing technology, the matrices are primarily affected by the state of water. The processes of phase transition, in particular, the moment of nucleation and the quality of frozen products are interdependent. The size of ice crystals is crucial for the final quality of frozen products, as they can damage the cell structure, affecting the product texture and color.

### 2.1 Freezing and ice nucleation

The freezing process has three stages: cooling to the freezing point, phase change with water crystallization, and tempering to the final temperature (9, 10). Crystallization consists of nucleation, whereby a thermodynamically stable critical nucleus forms, followed by diffusion-driven crystal growth (11).

Primary nucleation occurs in solutions without pre-existing crystals and can be homogeneous, arising spontaneously in a supercooled liquid, or heterogeneous, triggered by solid impurities (12, 13). Secondary nucleation happens when existing crystals induce new nuclei or break to create new nucleation surfaces (14). Nucleation determines crystal size and distribution, which is essential for uniform freezing and product quality (15–17).

Induced nucleation narrows the temperature range during freezing, yields smaller ice crystals, and shortens freezing time, reducing energy use (18–21). Chemical and biological methods modulate supercooling and increase nucleation temperature (22). Physical approaches also include cooling-rate control and high-pressure shift freezing, where rapid pressure release promotes supercooling and uniform nucleation (23).

### 2.2 Challenges related to freezing functional foods

Freezing functional foods disrupts cell structure and alters bioactive compounds, affecting texture, vitamin stability, PUFAs and antioxidants. Cell rupture increases exposure to oxidative enzymes and accelerates antioxidant loss (24). Freezing fresh fruits, vegetables, berries, and leafy greens is particularly challenging, as they contain both a high water content and a high proportion of the bioactive compounds listed above. For example, freezing nectarines (−80 °C, 15 min; then −18 °C, 30 days) reduced Trolox equivalent antioxidant capacity via polyphenol-oxidase activity and phenol loss (25); conventional freezing lowered ascorbic acid, anthocyanins and phenolics in red radish by 35.1, 24.1 and 19.2%, respectively (26). In *Agaricus bisporus* vitamins generally decreased, while *β*-carotene and lycopene were most resistant (27); vitamin C losses vary ~10 to 80% by species and conditions (28). Minerals are mostly stable, though iron, magnesium and calcium may drop in some frozen vegetables (29, 30). PUFAs degrade more at higher freezing temperatures, raising peroxide values and reducing omega-3 fatty acids (31). Freezing also alters amino-acid profiles (increased free amino acids and losses of some essential residues), with greater damage at slower cooling rates (32). Probiotics are especially vulnerable to oxidative, osmotic and ice-related damage; viability improves with cryoprotectants, encapsulation, strain selection and optimized freezing rates (33–36).

Considering the above aspects of freezing functional products, modified freezing methods incorporating additional technologies are of particular interest. Such approaches enable better preservation of food products during freezing, storage, and thawing.

## 3 Effects of physical field treatment on freezing parameters

Novel freezing technologies increasingly rely on physical fields and are being considered as advanced strategies for improving freezing efficiency and food quality (37).

### 3.1 Ultrasound-assisted freezing

**Figure 1:**
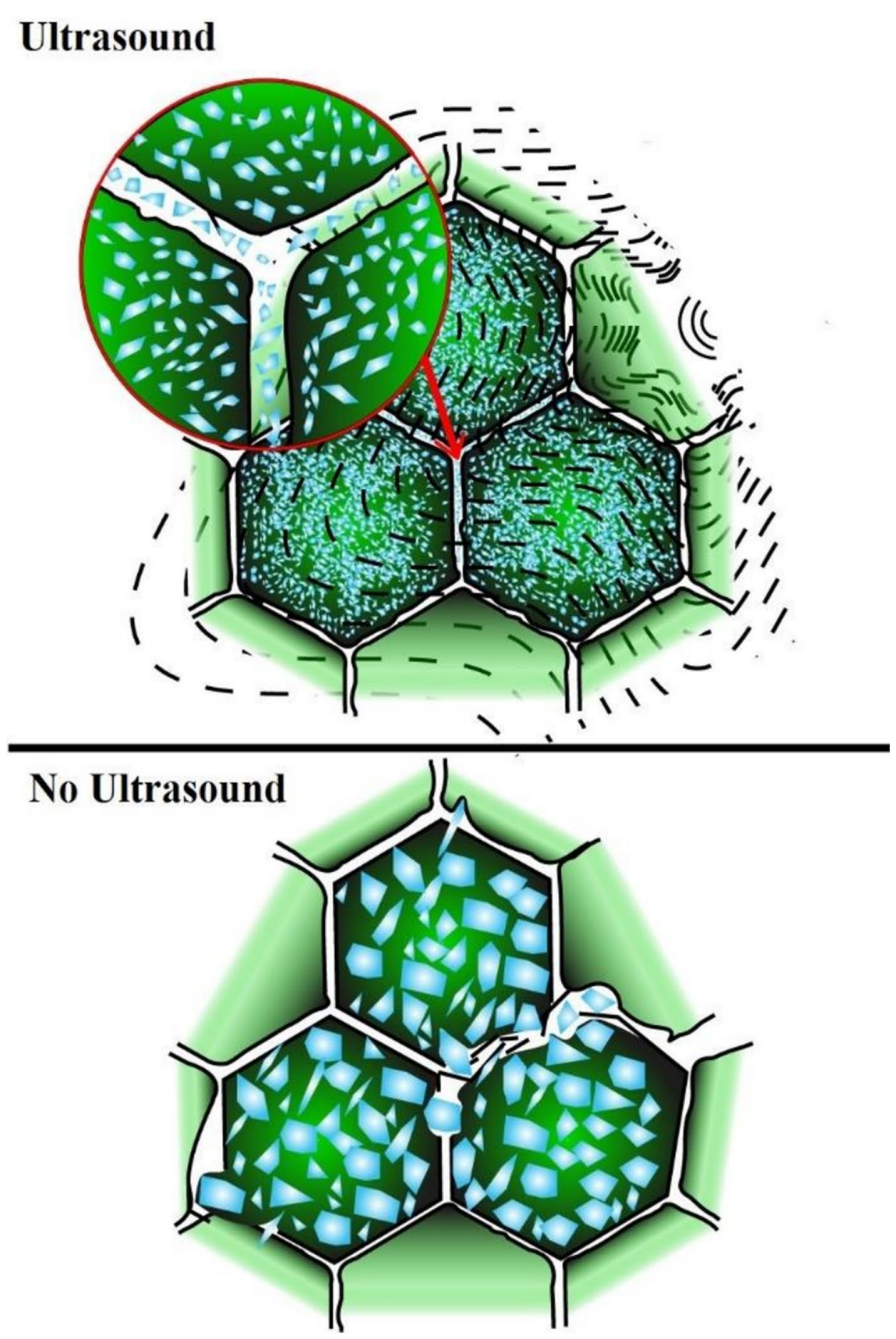
Effect of ultrasound assisted freezing on crystal size and cellular integrity.

Ultrasound-assisted freezing (UAF) has been studied more extensively than electromagnetic-assisted freezing. Ultrasound propagates through alternating compression and rarefaction cycles (38), with cavitation and microstreaming as the primary mechanisms of action (39). High-power ultrasound generates cavitation bubbles that collapse into stable or transient cavitation (40), while microstreaming produces strong eddy currents that enhance chemical and physical processes, including degassing and membrane disruption (38). Ultrasound treatment leads to the formation of smaller ice crystals due to initiation of multiple nuclei, preserving cell integrity (Figure 1).

During immersion freezing of potato slices, periodic ultrasound from 0 to −7 °C resulted in significantly faster freezing, reduced extracellular voids, and minimal cell disruption (39). Studies on melon and pineapple (41, 42) showed formation of microscopic channels and increased intercellular spaces, improving water diffusion and frozen quality. Ultrasound also affects enzymatic activity. Ultrasound reduced thawing losses in broccoli at 30 kHz, 0.35 W cm^−2^ (43), and in carp at 0.08 W g^−1^, 30 kHz (44). Similar improvements were reported in potatoes (20–40 kHz, 270 W) by (30). In frozen dairy and functional products, ultrasound produces smaller ice crystals and more air bubbles, improving texture and mouthfeel (36, 39, 45). In meat systems, ultrasound intensities of 0.2–0.6 W cm^−2^ reduced thawing time by up to 87% while maintaining texture (46). It was also reported that such intensities (25 kHz; 0.2–0.4 W cm^−2^) did not impair chemical or microbiological attributes during thawing (47). Ultrasound can be easily transmitted into frozen tissues, accelerating thawing without dehydration or overheating, and can be combined with water immersion using minimal specialized equipment (30, 48, 49).

### 3.2 Magnetic, electric and electromagnetic field assisted freezing

Magnetic fields disrupt water’s molecular equilibrium, enabling supercooling and resulting in rapid crystallization into fine ice crystals upon removal. Electric fields lower the nucleation energy barrier, initiating many nucleation sites and producing uniform ice with improved structural integrity. Both mechanisms control ice formation by altering water’s organization, leading to smaller crystals and better quality in frozen materials (7, 8, 50).

#### 3.2.1 Magnetic field assisted freezing

Several studies have demonstrated that magnetic treatment during freezing reduces moisture and water-soluble substance losses during thawing (51–54), decreases ice crystal size (55–57), and improves the quality parameters of bread made from frozen dough (58). Additionally, magnetic field treatment of vegetables prior to refrigerated storage influences enzymatic activity and contributes to the preservation of product quality (59, 60). Efficiency strongly depends on product type (61). A study (62) showed that magnetic treatment of deionized water produced a less pronounced supercooling effect than a 0.9% NaCl solution, indicating dependence on ionic concentration. Field type matters as well, as alternating magnetic fields in cherries increased crystallization temperature and prolonged phase transition, while constant fields accelerated crystallization without markedly altering the crystallization temperature (56).

#### 3.2.2 Electric field assisted freezing

Particular attention in the food industry is given to the application of pulsed electric fields, as their use as a pretreatment technology helps preserve such quality attributes as texture, flavor, functionality, and nutritional value by accelerating freezing rate and reducing ice crystal size (63, 64). Static electric fields also influence nucleation kinetics. Application of such fields is an effective tool for controlling crystallization in extracts, e.g., mushroom extract while in whole mushrooms it reduces drip loss and accelerates freezing (65). However, the implementation of electric fields in food freezing requires precise optimization of field intensity. It must be sufficiently strong to promote the formation of a fine and uniform ice structure, yet not exceed the threshold at which the risk of product damage increases and energy consumption becomes excessive (66).

Simultaneous magnetic and electric field application enhances cryopreservation efficiency by accelerating freezing, generating a fine, uniform ice network, reducing cellular injury and improving nutritional retention (67).

#### 3.2.3 Electromagnetic field assisted freezing

Currently, electromagnetic radiation (ER) has been reported to be used in four ways during freezing: (i) pretreatment with MW before freezing (68); (ii) continuous use of ER energy during freezing (69, 70); (iii) partial use of ER during freezing (10, 71); (iv) ER-assisted thawing (72, 73). Although the exact mechanism underlying ER-aided freezing has not been explained yet, current explanations rely on a limited set of working hypotheses.

MW-induced localized heating partially melts large crystals, which refreeze into smaller ones. MW application has been shown to enhance vitrification at ultralow temperatures (53). Early MW-assisted freezing work in the 1990s demonstrated MW effects during cryopreservation in liquid nitrogen using cryoprotectants (68, 69). A patented device also used MW to suppress spontaneous nucleation in supercooled liquids (21).

Application of pulsed MW energy in food, e.g., to pork tissue showed that higher MW power reduced ice crystal size and supercooling by 62 and 92%, respectively, whereas lower power produced larger crystals (74, 75). Increased MW power, however, also generated heat, slowing freezing. Comparable results were reported under radiofrequency-assisted freezing, which reduced structural damage in meat (71). Both pulsed and continuous MW reduced ice crystal size by about 25%, and longer MW exposure (at constant power) further decreased crystal size (76).

However, it has been demonstrated that the combination of a static magnetic field and a pulsed electric field did not reduce the overall freezing time of saline solutions and meat samples, highlighting the need for precise adjustment of electromagnetic treatment parameters to the specific food matrix and equipment design (77).

## 4 Effect of physical field treatment on the final quality of frozen functional foods

Most bioactive compounds remain stable at low temperatures, with degradation in frozen products mainly caused by oxidative stress and drip loss during thawing. Large ice crystals disrupt cellular integrity, however field-assisted freezing can reduce tissue rupture and oxidative stress, improving retention of functional ingredients. Table 1 summarizes the impact of physical field treatment on preserving functional food ingredients.

**Table 1:** Effect of field-assisted freezing on the stability of functional ingredients.

Functional ingredient	Food product	Physical-field settings	Optimal setting	Minimum effective setting	Maximum tested setting	References
UAF						
L-ascorbic acid, anthocyanins and polyphenolic compounds	Red radish	Frequency = 20 kHz; duty = 30 s on/30 s off	0.26 W cm^−2^	<0.17 W cm^−2^ weak nucleation	≥0.37 W cm^−2^ higher drip loss	(26)
–	Porcine longissimus muscles	Frequency = 30 kHz; Duty = 30 s on/30 s off; Duration = 8 min	180 W	120 W big crystals	300 W excessive heat generation	(99)
L-ascorbic acid, polyphenolic compounds	Potatoes	Frequency = 20 kHz (SUF), 20 + 28 kHz (DUF), 20 + 28 + 40 kHz (TUF); Duty = 30 s on/30 s off; Duration = 120 s	270 W (TUF)	270 W (SUF) weaker effect	–	(30)
L-ascorbic acid	Kiwi (*Actinidia deliciosa*)	Frequency = 20 kHz; Duration = 30 min	120 W	0 W no effect	300 W reduced ascorbic acid	(92)
L-ascorbic acid	Lotus (*Nelumbo nucifera*) root	Frequency = 30 kHz; Duty = 30 s on/30 s off; Duration = 6 min	150 W	90 W weaker effect	210 W vitamin C retention decreased	(93)
US + static magnetic field (SMF)						
Anthocyanin, total phenolic and flavonoid contents	Blueberries	US pretreatment: Frequency = 50 kHz; Duration = 5 min; SMF: Strength = 10 mT	200 W pretreatment +10 mT	200 W ultrasound pretreatment + 0 mT	200 W ultrasound pretreatment + 10 mT	(80)
EF + SMF						
L-ascorbic acid	Broccoli and cauliflower	EF: Intensity = 1–5 kV cm; SMF: Strength = 2–8 mT	Cauliflower: 3 kV cm; Broccoli: 5 kV cm	1 kV cm, 2 mT	5 kV cm, 8 mT	(79)
Pulsed electric field (PEF) + SMF						
Total anthocyanin, phenolic and ascorbic acid content	Strawberries	PEF: Strength = 40 kV; Duration = 30 min; SMF: Strength = 10 mT	PEF 40 kV, 30 min + SMF 10 mT	PEF 40 kV	–	(100)

Based on experimental data, it can be concluded that an ultrasound intensity of 0.26–0.27 W cm^−2^ at a frequency of 20 kHz with a 30 s on/30 s off allows acceleration of the freezing process while preserving functional ingredients. Nevertheless, the optimal parameters strongly depend on the properties of the treated material as well as on the type of equipment used. Accordingly, contradictory results have been reported for different products. For example, a study on apples (54) revealed no significant differences in the retention of phenolic compounds after ultrasound treatment at frequencies of 21 and 45 kHz, whereas a study on parsley leaves (78) showed that ultrasound treatment at 35 kHz led to greater losses than at 21 kHz. In addition, the simultaneous application of multiple ultrasound frequencies has been reported to improve the preservation of bioactive compounds (30).

A comparison of the effects of electric and magnetic fields on vegetable freezing showed that electric field treatment results in higher product quality. In particular, cauliflower subjected to electric field treatment exhibited lower juice loss, higher ascorbic acid content, and reduced cellular damage. Although both treatments improved the quality of frozen vegetables, electrostatic field treatment, specifically at a field strength of 3 kV cm^−1^, produced the best results (79). Combination of ultrasound pretreatment and static magnetic field freezing improved preservation of blueberry bioactive components by 33.67, 29.14, and 18.65% for anthocyanin, total phenolic and flavonoid contents, respectively (80).

There appears to be a lack of studies regarding the effect of field-assisted freezing on the viability of probiotic bacteria. The process of freezing bacteria cells is similar to those occurring in plant and animal tissues as they are also sensitive to freezing rates. Cell death may result from dehydration at slow freezing rates or intracellular ice formation at faster rates. Increasing freezing speed reduces crystal size, but thawing rate is also critical as during slow thawing, small intracellular crystals may merge into larger ones and damage probiotic bacteria cells (81). It has been reported that ultrasound can directly affect probiotic viability during freezing. In a study on *Lactobacillus plantarum*, power ultrasound at 25 kHz and 0.25 W cm^−2^ applied for 3 s induced controlled nucleation near −2 to −4 °C and significantly increased cell viability compared to non-irradiated samples, whereas irradiation at lower nucleation temperatures (≤ − 6 °C) reduced viability due to accelerated phase transition and intracellular ice formation. Ultrasound applied during the phase change stage for 4 min further improved viability while shortening freezing time. Ultrasound and electromagnetic treatments may promote more uniform formation of small ice crystals, potentially improving microbial survival during freezing, storage, and thawing, but this requires further experimental proof.

Of the freezing intensification methods, ultrasound is the most scientifically validated and scalable, though its industrial application is limited by high energy demands and signal attenuation in viscous products. Electric fields show moderate but variable effectiveness, constrained by safety risks and diminished performance in conductive materials. In contrast, magnetic and electromagnetic methods lack reliable scientific substantiation and face prohibitive economic barriers, making them impractical for industrial adoption.

Current research does not allow a clear separation of freezing parameters that preserve structure from those that degrade bioactive compounds. Outcomes depend heavily on the compound’s chemistry: water- and fat-soluble vitamins (e.g., ascorbic acid vs. *β*-carotene) differ in oxygen sensitivity, thermostability and interactions with released enzymes, so they respond differently to the same field-assisted protocol. Scale factors - product volume, equipment capability and exposure duration - change system thermodynamics and mass transfer, further complicating optimization. For multicomponent functional ingredients, each constituent may require its own parameter window, therefore, optimal freezing conditions must be determined individually for each product formulation.

## 5 Limitations of electromagnetic and ultrasonic treatment

Application of ultrasound or electromagnetic treatments in functional food products can affect the stability of certain bioactive components. This is supported by studies on ultrasound and electric-field processing of foods conducted without subsequent freezing (82, 83). While some compounds tend to be preserved better after treatment, others (especially L-ascorbic acid) may degrade due to such exposure (84). Cavitation during ultrasound treatment leads to the formation of -OH radicals, which can react with antioxidants and ascorbic acid, thereby reducing their content in the product (85–87). Some studies show that vitamin C underwent near-total degradation under any processing power (84, 88, 89). Increasing treatment duration and ultrasound intensity has also been shown to reduce ascorbic acid content (85) and total phenolics (90, 91) However, such risks are mainly associated with ultrasound powers above 150 W and prolonged exposure times exceeding 10 min, which are not representative of the comparatively low intensities and short treatment durations typically applied during freezing processes (92–94). Nevertheless, when using physical field treatment for functional foods, it is crucial to identify the optimal technological parameters that will not damage the desired functional components in the product.

## 6 Summary and outlook

This study presents an analysis of the available data on field-assisted freezing and its effects on the biologically active components of products. Research and reviews on field-assisted freezing of many regular food products have already been conducted, indicating that this topic is relevant and actively developing. The available data demonstrate the positive impact of these technologies on the quality of frozen products. On the other hand, preserving the functional properties of frozen foods has been less explored, despite the growing interest in functional foods. The primary mechanism for preserving bioactive compounds in functional products during field-assisted freezing is the maintenance of product structure, which retains these compounds within the intracellular space and reduces their losses due to leakage and oxidative stress. At the same time, some effects, such as the ultrasound-induced release of phenolic compounds, may have both positive and negative impacts, depending on the intended application, which highlights the need for a balanced technological strategy.

A comparative analysis of modern physical methods for intensifying food freezing reveals substantial differences in their mechanisms of action and effectiveness in influencing crystallization. Ultrasonic treatment (20–100 kHz) effectively creates fine, uniform ice crystals via acoustic cavitation, minimizing cellular damage. Electric fields (0.5–10 kV cm^−1^) reduce nucleation energy by aligning water dipoles, but their efficacy depends on product conductivity and electrode contact. Magnetic fields (1–100 mT) theoretically slow crystallization by influencing water clusters, though results are inconsistent, while microwaves are generally counterproductive due to their heating effect. Thus, ultrasound stands out as the most proven and direct technology for managing ice nucleation, whereas electric and magnetic fields represent promising yet less universal or incompletely understood approaches that require further investigation for industrial implementation. However, the ability of physical fields for improving the quality of frozen food products is highly dependent on the duration of exposure, the intensity, the frequency and other parameters. This means that more fundamental research of the underlying phenomena is still needed to establish the relevance of field-assisted freezing parameters for the technique’s efficiency.

An alternative to employing field-assisted freezing of functional foods could involve the application of encapsulation techniques, which are well-established and frequently used in the production of functional food products (95–98). These techniques can isolate the target bioactive compound from the external product environment, thereby reducing the influence of the aforementioned factors that promote bioactive degradation during storage thus they may be promising solutions. Nevertheless, further studies on frozen functional foods containing encapsulated bioactives are required.
